# Expression of Genes Related to Anti-Inflammatory Pathways Are Modified Among Farmers’ Children

**DOI:** 10.1371/journal.pone.0091097

**Published:** 2014-03-06

**Authors:** Remo Frei, Caroline Roduit, Christian Bieli, Susanne Loeliger, Marco Waser, Annika Scheynius, Marianne van Hage, Göran Pershagen, Gert Doekes, Josef Riedler, Erika von Mutius, Felix Sennhauser, Cezmi A. Akdis, Charlotte Braun-Fahrländer, Roger P. Lauener, as part of the PARSIFAL study team

**Affiliations:** 1 Swiss Institute of Allergy and Asthma Research (SIAF), University of Zurich, Davos, Switzerland; 2 Christine Kühne-Center for Allergy Research and Education, Zurich, Switzerland; 3 Children’s Hospital, University of Zurich, Zurich, Switzerland; 4 Swiss Tropical and Public Health Institute, Basel, Switzerland; 5 University of Basel, Basel, Switzerland; 6 Translational Immunology Unit, Department of Medicine Solna, Karolinska Institute and University Hospital, Stockholm, Sweden; 7 Clinical Immunology and Allergy Unit, Department of Medicine Solna, Karolinska Institute and University Hospital, Stockholm, Sweden; 8 Institute of Environmental Medicine, Karolinska Institutet, Stockholm, Sweden; 9 Institute for Risk Assessment Sciences, Utrecht University, Utrecht, The Netherlands; 10 Children’s Hospital Schwarzach, Schwarzach, Austria; 11 Dr. von Hauner Children’s Hospital, Ludwig Maximilians University Munich, Munich, Germany; 12 Kantonsspital St.Gallen, St.Gallen, Switzerland; Université Libre de Bruxelles, Belgium

## Abstract

**Background:**

The hygiene hypothesis states that children exposed to higher loads of microbes such as farmers’ children suffer less from allergies later in life. Several immunological mechanisms underpinning the hygiene hypothesis have been proposed such as a shift in T helper cell balance, T regulatory cell activity, or immune regulatory mechanisms induced by the innate immunity.

**Objective:**

To investigate whether the proposed immunological mechanisms for the hygiene hypotheses are found in farmers’ children.

**Methods:**

We assessed gene expression levels of 64 essential markers of the innate and adaptive immunity by quantitative real-time PCR in white blood cells in 316 Swiss children of the PARSIFAL study to compare farmers’ to non-farmers’ expressions and to associate them to the prevalence of asthma and rhinoconjunctivitis, total and allergen-specific IgE in serum, and expression of Cε germ-line transcripts.

**Results:**

We found enhanced expression of genes of the innate immunity such as IRAK-4 and RIPK1 and enhanced expression of regulatory molecules such as IL-10, TGF-β, SOCS4, and IRAK-2 in farmers’ children. Furthermore, farmers’ children expressed less of the T_H_1 associated cytokine IFN-γ while T_H_2 associated transcription factor GATA3 was enhanced. No significant associations between the assessed immunological markers and allergic diseases or sensitization to allergens were observed.

**Conclusion:**

Farmers’ children express multiple increased innate immune response and immune regulatory molecules, which may contribute to the mechanisms of action of the hygiene hypothesis.

## Introduction

The hygiene hypothesis was proposed on the observation that children with several siblings were at lower risk of developing rhinoconjunctivitis and atopic sensitization. The protective effect was assigned to more frequent infections during childhood [1]. Other findings supported this hypothesis, such as early attendance of a day-care nursery had a protective effect against the development of allergies and Italian military students with antibodies to hepatitis A virus showed a lower prevalence of atopy and atopic respiratory diseases [2], [3].

It was not only infections that seemed to protect children against allergies; in addition, the effect of rural lifestyle has been suggested as one of the major preventive factors for allergy development [4]. Especially, early life or prenatal contact to farm animals and consumption of non-pasteurized milk, were found to be associated with lower prevalence of allergies [5], [6], [7], [8], [9]. Although there were reports that didn’t observe a difference between farmers’ and rural control children [10], [11], the protective effect of a farm was assigned to exposure to higher loads and a broader diversity of bacterial and fungal components [12], [13]. Farmers’ children had reduced allergen-specific serum IgE levels and their blood leukocytes secreted less inflammatory cytokines in response to bacterial components and expressed more Toll-like receptors (TLR) [5], [12], [14], [15], [16]. Enhanced TLR expression at birth was associated with a lower risk to develop atopic dermatitis later in life [9].

As an immunological basis for the hygiene hypothesis, several mechanisms have been proposed including a shift in T helper cell type (T_H_)1/T_H_2 balance or alteration of dendritic cell, innate immunity and T regulatory cell (T_REG_) activities [17], [18]. The innate immunity is the origin of a T helper cell response [19] and the activation of this system is mediated via pathways activated via pattern recognition receptors (PRR) such as the toll-like receptor (TLR) signaling cascade or the nucleotide-binding oligomerization domain (NOD) signaling (Figure S1 in File S1). To provide proper homeostasis of the innate immune response, a complex regulatory network has evolved [20], [21]. In brief, after ligand binding, adaptor proteins such as myeloid differentiation primary response gene 88 (MyD88), toll-interleukin 1 receptor domain containing adaptor protein (TIRAP), or toll-like receptor adaptor molecule 1(TRIFF) are recruited to the receptor leading to activation of the kinases interleukin-1 receptor-associated kinase (IRAK)1, IRAK2, and IRAK4. These kinases activate TNF receptor-associated factor (TRAF) 6 leading to the translocation of transcription factors into the nucleus and to the activation of mitogen-activated protein (MAP) kinases [22]. Various negative regulators of the TLR signaling cascade have been described. The adaptor protein toll interacting protein (TOLLIP) keeps the cascade in a quiescent state before activation, while the non-functional kinase IRAK3, but also suppressors of cytokine signaling (SOCS)-1 and SOCS-3 are known to act as negative feedback inhibitors [23], [24].

Cytokines and co-stimulatory molecules of the innate immunity guide T helper cell activation and differentiation (Figure S1 in File S1) [25]. T_H_1 response is associated with inflammation and autoimmunity [26], the T_H_2 response with IgE-mediated allergy [27]. T_REG_ cells confer suppressive activity on T_H_1 and T_H_2 [28], [29], [30], [31].

Immunoglobulin class switching recombination (CSR) exchanges the constant region of the antibody. It is induced either T helper cell-dependent through interaction of the CD40 ligand (CD40L) with CD40 on B cells or innate immunity-dependent without contribution of T helper cells through B lymphocyte stimulator protein (BAFF) and a proliferation-inducing ligand (APRIL) (Figure S1 in File S1) [32], [33], [34]. Cytokines such as IFN-γ or IL-4 determine the kind of isotype the constant region of the antibody switches to through induction of transcription of so called germ-line transcripts (GLT). GLT make the DNA accessible for the recombinase AICDA (activation-induced cytidine deaminase) making the CSR [35], [36], [37].

The goal of the PARSIFAL (Prevention of Allergy Risk factors for Sensitization In children related to Farming and Anthroposophic Lifestyle) study was to identify potential factors from rural and anthroposophic lifestyles having a protective effect against allergies in children [5], [38]. Here, we assessed among the Swiss children of the PARSIFAL study [5], [6], [15] the gene expressions of essential molecules of the innate and the adaptive immunity (Table S1 and Figure S1 in File S1) and their association with farm-life and allergic diseases such as asthma and rhinoconjunctivitis, immunoglobulin CSR to IgE, and total or allergen-specific IgE in sera with the aim to better understand the immunological basis of the hygiene hypothesis using the example of farmers’ children.

## Methods

### Population and Questionnaires

We assessed the expression of relevant T helper cell marker genes and of genes of the innate immunity in the Swiss branch of the cross-sectional PARSIFAL study [5], [6], [15], [38]. EDTA blood samples were available from 140 farm and 176 reference children (Table 1). White blood cells were isolated immediately after blood sampling using the QIAmp RNA Blood Mini Kit (Qiagen, Hilden, Germany) and stored at −80°C. The questions on farming lifestyle and health outcomes were derived from the internationally validated International Study of Asthma and Allergies in Childhood II questionnaire and the Allergy and Endotoxin (ALEX) study, respectively, and it was validated for farmers’ and non-farmers’ children [38], [39], [40], [41], [42]. Children with reported doctor-diagnosed asthma once or obstructive bronchitis more than once in their lifetime were defined as having asthma ever. Rhinoconjunctivitis was defined by reported doctor diagnosis of allergic rhinitis ever. Mother or father atopic sensitization was defined as ever having asthma or rhinoconjunctivitis. The study was approved by the ethical review committee of Basel and written informed consent was obtained from all parents.

**Table 1:** Characteristics of the children.

	All	N = 316	Farmer (44.3%)	N = 140	Non-Farmer (55.7%)	N = 176	p-value*
	%	N	%	N	%	N	
**Gender**							
**Girl**	49.1	155	47.9	67	50.0	88	0.705
**Age**							
**5–6 yrs**	11.1	35	10.0	14	11.9	21	0.376
**7–8 yrs**	27.5	87	25.0	35	29.6	52	
**9 yrs**	17.4	55	16.4	23	18.2	32	
**10–11 yrs**	26.0	82	25.7	36	26.1	46	
**12–13 yrs**	18.0	57	22.9	32	14.2	25	
**Mother atopic sensitization**	19.9	63	10.7	15	27.3	48	<0.001
**Father atopic sensitization**	14.2	45	5.0	7	21.6	38	<0.001
**Asthma**	7.3	23	3.6	5	10.2	18	0.024
**Rhinoconjunctivitis**	5.7	18	2.9	4	8.0	14	0.052
**Atopic sensitization, ≥0.35 kU/L**	31.9	100/314	21	29	40.3	71	<0.001

*Chi-square test farmer vs non-farmer.

Mother or father atopic sensitization was defined as ever having asthma or rhinoconjunctivitis.

Children with reported doctor-diagnosed asthma once or obstructive bronchitis more than once in their lifetime were defined as having asthma ever.

Rhinoconjunctivitis was defined by reported doctor diagnosis of allergic rhinitis ever.

Atopic sensitization was assessed for 314 of the 316 children.

### IgE Serology

Atopic sensitization was indicated if the child had at least one allergen-specific serum IgE result of ≥0.35 kU/L against common inhalant allergens (ImmunoCAP System, Thermo Fisher Scientific/Phadia AB, Uppsala, Sweden: birch, timothy, mugwort, *Dermatophagoides pteronyssinus* and *farinae*, cat-, dog-, and horse epithelium, and *Cladosporium herbarum*) and/or food allergens (Fx5: egg white, milk, fish, wheat, peanut, and soy) [43]. Total IgE was assessed using the ImmunoCAP System and the cutoff was 2 kU/L.

### RT-PCR and Quantitative Real-time PCR (TaqMan)

Total RNA was isolated from white blood cells using the QIAmp RNA Blood Mini Kit (Qiagen, Hilden, Germany) supplemented with RNase-free DNase (Qiagen) and stored at minus 80°C [5], [15]. For reverse transcription (RT) of RNA we used 300 ng of total RNA in a final volume of 30 µl and added adequate amounts of TaqMan Reverse Transcription Reagents (Applied Biosystems, Foster City, USA). The quantification of Cε germline transcripts we used the ABI Prim 7700 Sequence Detection System (Applied Biosystems) and the following primers: forward 5′-ACAGGCACCAAATGGACGAC-3′, reverse 5′-TTGCAGCAGCGGGTCAA-3′. The minor groove binding probe had the sequence 5′-CACAGAGCCCATCCG-3′. The quantification of the other genes was performed on an ABI Prism 7900 Sequence Detection System (Applied Biosystems) using the TaqMan low density array (LDA) system of Applied Biosystems. The determined gene expression values were normalized to the parallel measured endogenous controls 18S rRNA. We analyzed the data with the comparative Ct method according to the manufacturer’s instructions (Applied Biosystem). Tests regarding RNA stability were performed [44].

### Statistical Analysis

Data analysis was conducted using SAS software version 9.2 (SAS Institute). Differences in characteristics of children regarding farming status were tested by Chi2-test and expressed as P-values. Because the distribution of the gene expression levels were skewed, these variables were log transformed values (natural logarithm), resulting in a good approximation to the normal distribution (data not shown). With measurements of gene expression with more than 12% of undetectable, we used binomial variables for these gene expression levels, divided by the median. Multiple linear regressions were used to investigate the association between farmer status and gene expression levels. Geometric means ratios (GMRs) and 95% confidence interval (CI) were reported. Adjustment was made for the following potential confounder: gender, age of the child, atopic sensitization of the mother and of the father. The number of siblings and passive smoking were added in the model, but did not change the results, so they were not kept in the final model. Logistic regressions were used to analyze the association between gene expression levels and asthma, rhinoconjunctivitis, and atopic sensitization, adjusting for the same covariates as mentioned above. Results were expressed in odds ratios (ORs) and 95% CIs. For the association with the levels of gene expression and total IgE antibody levels and CSR to IgE, linear regression models were performed, as both dependent variables are continuous and were log-transformed. Those associations were corrected for multiple testing using the false discovery rate (Benjamini procedure) (*P* value ≤0.05 was considered as significant) [45].

## Results

### Association of Farm Life with Gene Expression of TLR Co-receptors, Molecules of the TLR Signaling Cascade, and of PRR

The study group was composed of 316 children between the ages of 5 to 13. 44.3% of them were farmers’ children (Table 1). Among farmers’ children a diagnosis of asthma (p = 0.024) or rhinoconjunctivitis (p = 0.052) and atopic sensitization (p<0.001) was lower compared to non-farmers’ – rural control children. The proportion of parents with atopic sensitization was also reduced among farmers’ children. We have previously shown that living on a farm was associated with an increased expression of some TLR in this study population [5], [15]. Here, we completed the set of PRR by measuring the gene expression of co-receptors of TLR, molecules of the TLR signaling cascade, and of PRR such as NOD1 and NOD2 or TREM1 and compared the expression of farmers’ children to non-farmers’ children (Table 2 and Table S2 in File S1). An increased gene expression significant for IRAK-2 and RIPK1 after correction for multiple testing and of borderline significance for TRIF, IRAK-1 and TBK1 was found among farmers’ children. Only the gene expression of IRAK-4 was significant decreased. Furthermore, the HLA-DRA gene was more expressed in farmers’ children. Of the negative regulators of the TLR signaling cascade, we identified the expression of SOCS-1 (borderline significance) and SOCS-4 genes to be increased in farmers’ children (Table 2).

**Table 2:** Association of farm life with gene expression of TLR signaling cascade.

	GMR*	95% CI		p-value	p-value after mutliple testing
**MYD88**	0.85	0.73	0.99	0.035	0.101
**TRIF**	1.21	1.04	1.41	0.015	0.056
**IRAK1**	1.17	1.07	1.29	0.001	0.052
**IRAK2**	**1.89**	**1.56**	**2.29**	**0.001**	**0.026**
**IRAK4**	**0.83**	**0.73**	**0.96**	**0.01**	**0.047**
**RIPK1**	**1.27**	**1.13**	**1.44**	**0.001**	**0.017**
**TBK1**	1.17	1.04	1.33	0.012	0.052
**HLA-DRA**	**1.54**	**1.29**	**1.84**	**0.001**	**0.013**
**SOCS1**	1.19	1.03	1.39	0.021	0.068
**SOCS4**	**1.17**	**1.04**	**1.31**	**0.008**	**0.042**

Boldface values are significant (p<0.05).

*adjusted for sex, age, mother atopic sensitization and father atopic sensitization.

GMR, geometric mean in farmers’ children compared with non-farmers’ children.

### Association of Gene Expression of PRR and Molecules of the TLR Signaling Cascade with Asthma, Rhinoconjunctivitis, CSR to IgE, and Total or Allergen-specific IgE

We wanted to know whether there was a correlation between gene expression of PRR, TLR co-receptors, and molecules of the TLR signaling cascade and the prevalence of asthma or rhinoconjunctivitis, CSR to IgE, and total or allergen-specific IgE in sera (Table 3 and Table S3 in File S1). To assess CSR to IgE, we measured the expression of Cε GLT by quantitative real-time PCR. We found that the expression of TLR9 and HLA-DRA genes was significantly positively associated with Cε GLT expression. Furthermore, we found that the expression of some molecules such as CD14, TLR5, TLR6, TLR8, TREM-1, but also IRAK-1 and IRAK-2 genes was significantly negatively associated with total serum IgE, atopic sensitization, or asthma. However, all this values lost significance after correction for multiple testing. IRAK-1, IRAK-2, and RIPK1 were the only molecules, whose gene expression was associated to both farming environment and asthma. In this study population, we observed a reduced risk of having asthma among farmers’ children compared to non-famers’ children (crude OR: 0.33; 95% CI: 0.12–0–90). In order to test the contribution of these molecules in this protective effect, we added these 3 molecules, as predictors in the logistic model and we could observe that the protective farming effect on asthma was partially reduced (adjusted OR: 0.67; 95% CI: 0.22–2.00).

**Table 3:** Association of PRR and molecules of the TLR signaling cascade with CSR to IgE.

	OR*	95% CI		P-value	p-value after mutliple testing
**TLR7**	1.33	1.13	1.57	0.001	0.063
**TLR9**	**1.37**	**1.16**	**1.61**	**0.001**	**0.032**
**TLR10**	0.90	0.83	0.98	0.017	0.089
**IRAK2**	1.23	1.04	1.45	0.014	0.080
**HLA-DRA**	**1.30**	**1.08**	**1.55**	**0.005**	**0.045**
**SOCS6**	1.14	1.03	1.27	0.012	0.095
**TRIAD3**	1.49	1.09	2.02	0.012	0.084

Boldface values are significant (p<0.05).

*adjusted for sex, age, mother atopic sensitization and father atopic sensitization.

OR, odds ratio of farmers’ children compared with non-farmers’ children.

### Association of Farm Life with Gene Expression of Markers of T Helper Cell Subtypes and Cytokines

Next, we investigated whether farm life resulted in skewing of T_H_1/T_H_2 balance or in induction of T_REG_ cells in children. Neither the expression of the T-bet gene, the transcription factor driving the phenotype of T_H_1, nor the expression of the chemokine receptor CCR5 gene showed an association with farm life (Table 4). Expression of the T_H_2 marker gene, the transcription factor GATA-3, was positively associated with being a farm child. No association was found with the level of expression of T_REG_ cell marker gene FOXP3.

**Table 4:** Association of farm life with gene expression of markers of T helper cell subtypes.

	GMR*	95% CI		p-value	p-value multiple testing
**T_H_1**					
**T-bet**	1.07	0.91	1.27	0.397	
**CCR5**	0.83	0.5	1.39	0.481	
**T_H_2**					
**GATA3**	**1.28**	**1.08**	**1.51**	**0.004**	**0.026**
**CCR3**	0.71	0.54	0.93	0.014	0.056
**CCR4**	1.27	1.01	1.6	0.037	0.101
**T_REG_**					
**FOXP3**	1.02	0.87	1.19	0.808	

Boldface values are significant (p<0.05).

*adjusted for sex, age, mother atopic sensitization and father atopic sensitization.

GMR, geometric mean in farmers’ children compared with non farmers’ children.

T helper cells exhibit their function via release of a specific cytokine pattern. We compared the gene expression of cytokines in blood leukocytes of farmers’ with non-farmers’ children (Table 5). Gene expression of the T_H_1-associated cytokine IL-12α was not affected by farm life. However, gene expression of INF-γ was significantly down-regulated in blood leukocytes of farmers’ children. Gene expression of the T_H_2-associated cytokine IL-4 was also decreased (borderline significance) in farmers’ children. The gene expression of regulatory cytokines such as IL-10 and TGF-β was highly induced among farmers’ children. Furthermore, farmers’ children expressed more of the TNF-α gene.

**Table 5:** Association of farm life with the expression of cytokine genes.

	GMR*	95% CI		p-value	p-value multiple testing
**IL12α**	0.93	0.63	1.38	0.721	
**IFN-γ**	**0.29**	**0.18**	**0.46**	**0.001**	**0.010**
**IL4** 1	0.87	0.77	0.97	0.015	0.052
**IL10**	**3.45**	**1.82**	**6.56**	**0.001**	**0.009**
**TGF-β**	**1.16**	**1.05**	**1.29**	**0.005**	**0.029**
**IFN-β** 1	0.96	0.86	1.07	0.465	
**IL1b**	1.32	0.98	1.78	0.072	
**IL6**	0.88	0.44	1.79	0.730	
**IL8**	1.13	0.84	1.54	0.419	
**IL18**	0.88	0.71	1.09	0.251	
**IL21** 1	0.98	0.88	1.08	0.648	
**TNF-α**	**1.43**	**1.21**	**1.69**	**0.001**	**0.007**

Boldface values are significant (p<0.05).

*adjusted for sex, age, mother atopic sensitization and father atopic sensitization.

GMR, geometric mean in farmers’ children compared with non farmers’ children.

1 Binomial values.

### Association of Gene Expression of Markers of T Helper Cell Subtypes and Gene Expression of Cytokines with Asthma, Rhinoconjunctivitis, CSR to IgE, and Total or Allergen-specific IgE

Moreover, we were interested whether T helper cell balance was associated with asthma, rhinoconjunctivitis, immunoglobulin CSR to IgE, and total or allergen-specific IgE in sera. We found a positive association between immunoglobulin CSR to IgE and the gene expression of transcription factors T-bet and GATA-3 (Table S4 in File S1). There was no association with gene expression of any of the transcription factors with total serum IgE, atopic sensitization, asthma, and rhinoconjunctivitis. However, we found a positive association of TNF-α gene expression with the expression of Cε GLT (Table S5 in File S1). There were no further significant associations between the expression of cytokine genes and asthma, rhinoconjunctivitis, and total or allergen-specific IgE.

### Association of Farm Life with Gene Expression of APRIL, BAFF, CD40L, and AICDA

We further were interested whether the expression of co-stimulatory molecules crucial in inducing CSR was associated with farm life. We measured the gene expression of APRIL, BAFF, CD40L, and AICDA in blood leucocytes of children and no association with farming status was observed (Table S6 in File S1).

### Association of Gene Expression of APRIL, BAFF, CD40L, and AICDA with Asthma, Rhinoconjunctivitis, CSR to IgE, and Total or Allergen-specific IgE

Finally, we investigated whether the expression of APRIL, BAFF, CD40L, and AICDA genes in blood leucocytes of children was associated with the prevalence of asthma and rhinoconjunctivitis, CSR to IgE, and total and allergen-specific IgE in sera. Only the expression of the Cε GLT was positively associated with CD40L gene expression (Table S7 in File S1).

## Discussion

In this study we show that farmers’ children have an increased expression of innate immunity and regulatory molecules, which may lead to changes in thresholds for the activation of an inflammatory immune response. However, no significant association between gene expressions and allergic diseases or atopic sensitization was shown. Investigations comparing farmers’ with urban children or taking into account the gut microbial flora, the nutrition, or infections might clarify this issue in more detail. Moreover, although our data showed no shift in T helper cell balance in farmers’ children, different levels of T helper cell-associated cytokines were observed. Additionally, immunoglobulin CSR to IgE was enhanced via T cell activation shown by strong positive associations between the expression Cε GLT and the expression of CD40L or the T helper cell transcription factors.

Although farmers’ children expressed more T_H_2 transcription factor GATA-3 and this was associated with enhanced switching to IgE, they were less sensitized what indicates other immunological mechanisms to be important. Our findings coincide with other observations that the molecular basis of allergic disorders cannot be explained by the T_H_2 paradigm [18], [46]. These observations include that IFN-γ, IL-17, and neutrophils are found in lungs of asthma patients and treatments targeting T_H_2 cells failed to be effective [47]. It is interestingly noted here that not only T_H_2-associated diseases have increased over the past decades in parallel with elevated hygiene conditions, but also T_H_1-associated inflammatory and autoimmune diseases [48], [49], [50]. There are patients with a coincidence of allergic and autoimmune disease [51]. Moreover, allergic diseases show a substantial autoimmune profile and T_H_1 response plays an important role in chronicity and tissue injury in atopic diseases [52], [53], [54]. Our results, combined with the observations described above, does not support the fact that skewing of T helper cell differentiation is an immunological mechanism for the hygiene hypothesis at least in school age children. In newborn children, the situation might be different. It has been shown that reduced IFN-γ secretion after ex-vivo re-stimulation of cord blood cells with phorbol 12-myristate 13-acetate was associated with reduced allergen-specific IgE [55]. The different results between newborn and school age children might be based on different development status of the immune system or on the different methods to assess the IFN-γ expression. It is important to note here that our data shows direct analyses of the cells without any cultures, representing a direct *in vivo* situation compared to cultures of cells for several days.

Although the present study did not allow us to investigate immunological mechanisms in deep or even to discover new mechanisms, we were able to reproduce mechanisms in humans that have been demonstrated in cell cultures or mice experiments. One important mechanism that we were able to reproduce was the following: we have previously shown that the expressions of some TLR genes such as TLR2, TLR4, and TLR8 were enhanced among the farmers’ children included in the study [5], [15]. Furthermore, the expression of the TLR signaling molecules IRAK-1 and RIPK1 were increased in farmers’ children. IRAK-1 deficiency in mice attenuates but does not eliminate TLR-induced NF-κB and MAPK activation and gene induction. So, activation of the innate immunity is still guaranteed. Interestingly, TLR4-mediated STAT3-dependent IL-10 induction is impaired in IRAK-1 deficient cells [22]. Therefore, we propose that higher TLR expression together with more IRAK-1 activity ends in higher IL-10 levels in farmers’ children. IL-10 has been shown to be the crucial regulatory cytokine of the immune system by inhibiting inflammatory cytokine production by the innate immunity and eliciting anergy in T cells [25]. Moreover, IL-10 plays a role in allergen-specific immunotherapy [56]. In our study, we were able to show a strong increase of gene expression of regulatory cytokines, IL-10 and TGF-β, among farmer’s children, but we were not able to associate those gene expressions directly with allergic disorders, CSR to IgE, or atopic sensitization. However, we observed a strong down-regulated expression of T_H_1 and T_H_2 associated cytokines INF-γ and IL-4 in farmers’ children that might be caused by enhanced IL-10 levels.

The cross-sectional design of the study, the mixed cell population in peripheral blood in which we performed the measurements and the limited amount of children reduced the significance of the study. Thus, we were not able to investigate more in detail the role of farm-related environmental factors such as animal contact or nutrition on the expression of the markers of the immune system. Furthermore, the mentioned limitation might be the reason for not finding significant associations to the health outcomes, asthma and rhinoconjunctivitis. Although we could show that the farm-environmental mediated up-regulation in IRAK-1, IRAK-2, and RIPK1 contributed partially to their reduced incidence of asthma. In order to better understand the farming environment effect on the maturation of the immune system and on the development of allergic diseases, longitudinal studies are needed.

In summary, the immune system of farmers’ children seems to comprise the same composition of T helper cell subtypes compared to non-farmers’ children, but farmers’ T helper cells might be in a more anergic status and therefore they secreted less cytokines. This anergic status might be caused by regulatory cytokines induced by the innate immunity.

## Supporting Information

File S1 Includes Figure S1 and Tables S1–S7. Figure S1, T helper cell differentiation and B cell activation by the innate immunity. Schematic overview, how microbes activate the innate immune systems leading to T helper cell differentiation and proliferation and how immunoglobulin class switching recombination is induced via a T helper cell-dependent and independent pathway. APC, antigen-presenting cell; red, gene expression of these marker molecules was measured in this study. Table S1, list of genes their expression was assessed. Table S2, association of farm life with gene expression of TLR co-receptors, molecules of the TLR signaling cascade, and of PRR. Table S3, association of PRR, TLR co-receptors, and molecules of the TLR signaling cascade with asthma, rhinoconjunctivitis, CSR to IgE, and total or allergen-specific IgE. Table S4, association of gene expression of markers of T helper cell subtypes with asthma, rhinoconjunctivitis, CSR to IgE, and total or allergen-specific IgE. Table S5, association of gene expression of cytokines with asthma, rhinoconjunctivitis, CSR to IgE, and total or allergen-specific IgE. Table S6, association of farm life with gene expression of APRIL, BAFF, CD40L, and AICDA. Table S7, association of gene expression of APRIL, BAFF, CD40L, and AICDA with asthma, rhinoconjunctivitis, CSR to IgE, and total or allergen-specific IgE.
(DOC)
