# Supplementary material for: Expression of Genes Related to Anti-Inflammatory Pathways Are Modified Among Farmers’ Children
Source: PLoS One. 2014 Mar 6;9(3):e91097. doi: 10.1371/journal.pone.0091097 (PMC3946278; doi:10.1371/journal.pone.0091097)
Supplement: File S1 — Includes Figure S1 and Tables S1–S7. Figure S1, T helper cell differentiation and B cell activation by the innate immunity. Schematic overview, how microbes activate the innate immune systems leading to T helper cell differentiation and proliferation and how immunoglobulin class switching recombination is induced via a T helper cell-dependent and independent pathway. APC, antigen-presenting cell; red, gene expression of these marker molecules was measured in this study. Table S1, list of genes their expression was assessed. Table S2, association of farm life with gene expression of TLR co-receptors, molecules of the TLR signaling cascade, and of PRR. Table S3, association of PRR, TLR co-receptors, and molecules of the TLR signaling cascade with asthma, rhinoconjunctivitis, CSR to IgE, and total or allergen-specific IgE. Table S4, association of gene expression of markers of T helper cell subtypes with asthma, rhinoconjunctivitis, CSR to IgE, and total or allergen-specific IgE. Table S5, association of gene expression of cytokines with asthma, rhinoconjunctivitis, CSR to IgE, and total or allergen-specific IgE. Table S6, association of farm life with gene expression of APRIL, BAFF, CD40L, and AICDA. Table S7, association of gene expression of APRIL, BAFF, CD40L, and AICDA with asthma, rhinoconjunctivitis, CSR to IgE, and total or allergen-specific IgE. (DOC) [file pone.0091097.s001.doc]

**Figure S1**

**
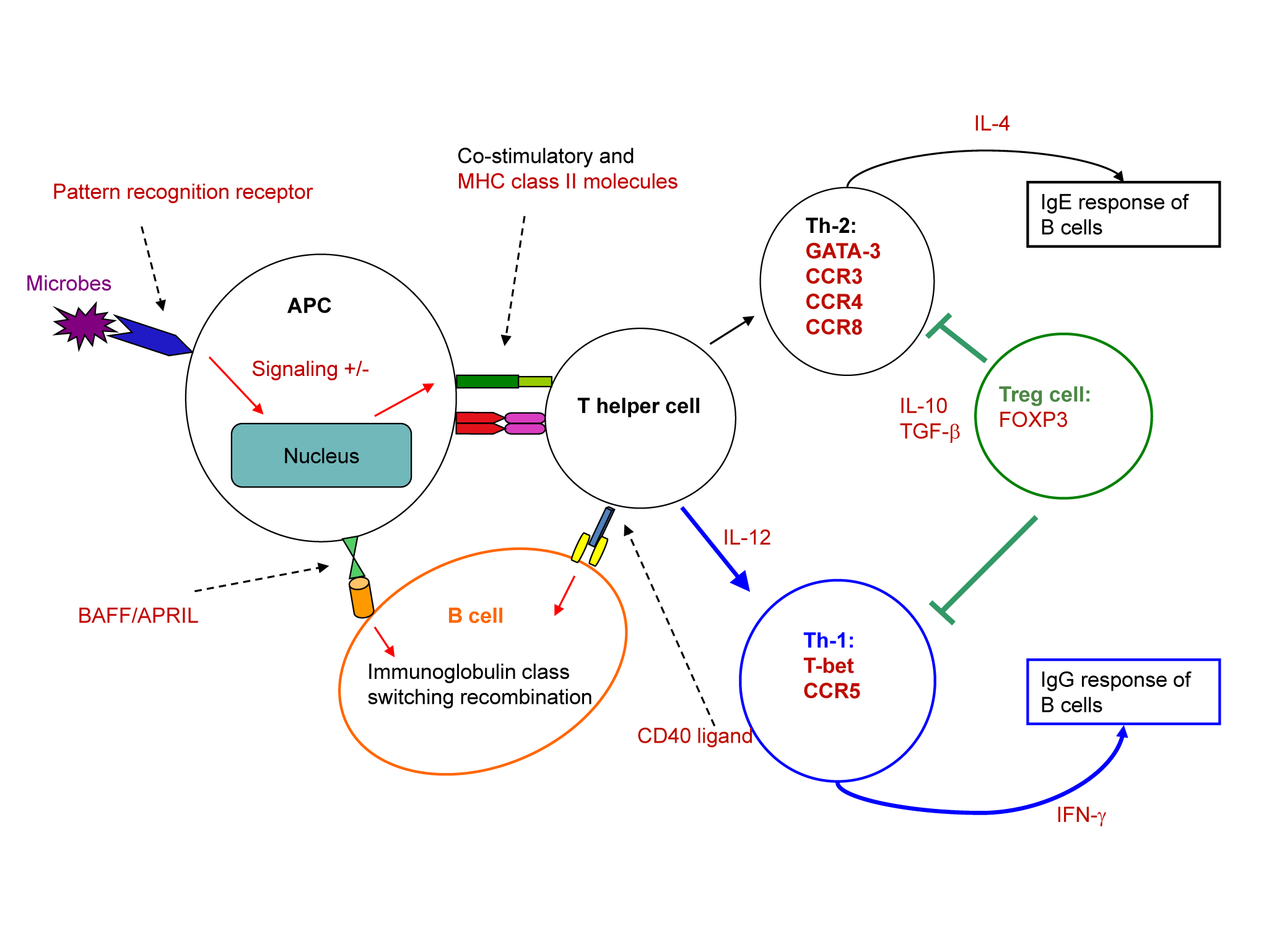
**

**Table S1. List of genes their expression was assessed.**

| Gene Name | Gene Symbol | Function | Reference | | |  | |  | |  |
| --- | --- | --- | --- | --- | --- | --- | --- | --- | --- | --- |
|  |  |  |  | | |  | |  | |  |
| PRR and TLR co-receptors |  |  |  | | |  | |  | |  |
| Toll-like receptor 1 | TLR1 | PRR | [1,2,3] | | |  | |  | |  |
| Toll-like receptor 2 | TLR2 | PRR | [1,2,3] | | |  | |  | |  |
| Toll-like receptor 3 | TLR3 | PRR | [1,2,3] | | |  | |  | |  |
| Toll-like receptor 4 | TLR4 | PRR | [1,2,3] | | |  | |  | |  |
| Toll-like receptor 5 | TLR5 | PRR | [1,2,3] | | |  | |  | |  |
| Toll-like receptor 6 | TLR6 | PRR | [1,2,3] | | |  | |  | |  |
| Toll-like receptor 7 | TLR7 | PRR | [1,2,3] | | |  | |  | |  |
| Toll-like receptor 8 | TLR8 | PRR | [1,2,3] | | |  | |  | |  |
| Toll-like receptor 9 | TLR9 | PRR | [1,2,3] | | |  | |  | |  |
| Toll-like receptor 10 | TLR10 | PRR | [1,2,3] | | |  | |  | |  |
| CD14 | CD14 | TLR co-receptor | [1,2,3] | | |  | |  | |  |
| Lymphocyte antigen 96 / MD-2 | LY96/MD-2 | TLR co-receptor | [1,2,3] | | |  | |  | |  |
| Triggering receptor expressed on myeloid cells 1 | TREM1 | PRR | [4] | | |  | |  | |  |
| Caspase recruitment domain family, member 15 | CARD15/NOD2 | PRR | [5,6] | | |  | |  | |  |
| Caspase recruitment domain family, member 4 | CARD4/NOD1 | PRR | [5,6] | | |  | |  | |  |
|  |  |  |  | | |  | |  | |  |
| TLR signaling cascade |  |  |  | | |  | |  | |  |
| Myeloid differentiation primary response gene (88) | MYD88 | Adaptor protein | [2,5,7] | | |  | |  | |  |
| Toll-interleukin 1 receptor (TIR) domain containing adaptor protein | TIRAP | Adaptor protein | [2,5,7] | | |  | |  | |  |
| TIR domain containing adaptor inducing interferon-beta | TRIF | Adaptor protein | [2,5,7] | | |  | |  | |  |
| TNF receptor-associated factor 6 | TRAF6 | Adaptor protein | [2,5,7] | | |  | |  | |  |
| Interleukin-1 receptor-associated kinase 1 | IRAK1 | Kinase | [2,5,7,8] | | |  | |  | |  |
| Interleukin-1 receptor-associated kinase 2 | IRAK2 | Kinase | [7,8] | | |  | |  | |  |
| Interleukin-1 receptor-associated kinase 4 | IRAK4 | Kinase | [2,5,7,8] | | |  | |  | |  |
| Protein kinase, interferon-inducible double stranded RNA dependent | PRKR | Kinase | [2,5,7] | | |  | |  | |  |
| Receptor (TNFRSF)-interacting serine-threonine kinase 1 | RIPK1 | Kinase | [2,5,7] | | |  | |  | |  |
| Receptor-interacting serine-threonine kinase 2 | RIPK2 | Kinase | [2,5,7] | | |  | |  | |  |
| TANK-binding kinase 1 | TBK1 | Kinase | [2,5,7] | | |  | |  | |  |
| Major histocompatibility complex, class II, DR alpha | HLA-DRA | Enhancer of TLR signaling | [9] | | |  | |  | |  |
|  |  |  |  | | |  | |  | |  |
| TLR signaling cascade - negative regulators |  |  |  | | |  | |  | |  |
| Suppressor of cytokine signaling 1 | SOCS1 | Inhibitor of JAK-STAT and TLR pathway | | [7] [10] | | | |  | |  |
| Suppressor of cytokine signaling 2 | SOCS2 |  |  | | |  | |  | |  |
| Suppressor of cytokine signaling 3 | SOCS3 |  |  | | |  | |  | |  |
| Suppressor of cytokine signaling 4 | SOCS4 |  | | | |  | |  | |  |
| Suppressor of cytokine signaling 5 | SOCS5 |  |  | | |  | |  | |  |
| Suppressor of cytokine signaling 6 | SOCS6 |  |  | | |  | |  | |  |
| Suppressor of cytokine signaling 7 | SOCS7 |  |  | | |  | |  | |  |
| Interleukin-1 receptor-associated kinase 3 | IRAK3/IRAK-M | Kinase negative | [7,8,11] | | |  | |  | |  |
| Toll interacting protein | TOLLIP | Adaptor | [7,12] | | |  | |  | |  |
| Sterile alpha and TIR motif containing 1 | SARM1 | Adaptor | [7] | | |  | |  | |  |
| TRIAD3 protein | TRIAD3 | Ligase promotes TLR4 and TLR9 degradation | [13] | | |  | |  | |  |
| Single Ig IL-1R-related molecule | SIGIRR | Regulator of TLR4 | | [14] | | | |  | |  |
| Receptor-interacting serine-threonine kinase 3 | RIPK3 | Kinase to regulate TRIF | [15] | | |  | |  | |  |
|  |  |  |  | | |  | |  | |  |
| T_H_1 associated |  |  |  | | |  | |  | |  |
| T-box 21 | T-bet | T_H_1 transcription factor | [16] | | |  | |  | |  |
| Chemokine (C-C motif) receptor 5 | CCR5 |  |  | | |  | |  | |  |
|  |  |  |  | | |  | |  | |  |
| T_H_2 associated |  |  |  | | |  | |  | |  |
| GATA binding protein 3 | GATA3 | T_H_2 transcription factor | [17] | | |  | |  | |  |
| Chemokine (C-C motif) receptor 3 | CCR3 |  |  | | |  | |  | |  |
| Chemokine (C-C motif) receptor 4 | CCR4 |  |  | | |  | |  | |  |
| Chemokine (C-C motif) receptor 8 | CCR8 |  |  | | |  | |  | |  |
|  |  |  |  | | |  | |  | |  |
| T_REG_ associated |  |  |  | | |  | |  | |  |
| Forkhead box P3 | FOXP3 | T_REG_ transcription factor | [18] | | |  | |  | |  |
|  |  |  |  | | |  | |  | |  |
| Cytokines |  |  |  | | |  | |  | |  |
| Interleukin 12 alpha | IL-12α |  | [19] | | |  | |  | |  |
| Interferon, gamma | IFN-γ |  | [19] | | |  | |  | |  |
| Interleukin 4 | IL-4 |  | [19] | | |  | |  | |  |
| Interleukin 10 | IL-10 |  | [19] | | |  | |  | |  |
| Transforming growth factor, beta | TGF-β |  | [19] | | |  | |  | |  |
| Interferon, beta | IFN-β |  | [19] | | |  | |  | |  |
| Interleukin 1, beta | IL-1β |  | [19] | | |  | |  | |  |
| Tumor necrosis factor (TNF superfamily, member 2) | TNF-α |  | [19] | | |  | |  | |  |
| Interleukin 6 | IL-6 |  | [19] | | |  | |  | |  |
| Interleukin 8 | IL-8 |  | [19] | | |  | |  | |  |
| Interleukin 18 | IL-18 |  | [19] | | |  | |  | |  |
| Interleukin 21 | IL-21 |  | [19] | | |  | |  | |  |
|  |  |  |  | | |  | |  | |  |
| CSR |  |  |  | | |  | |  | |  |
| tumor necrosis factor (ligand) superfamily, member 13 / APRIL | TNFSF13/APRIL | (T cell independent) |  | | |  | |  | |  |
| tumor necrosis factor (ligand) superfamily, member 13b / BAFF | TNFSF13B/BAFF | (T cell independent) |  | | |  | |  | |  |
| tumor necrosis factor (ligand) superfamily, member 5 / CD40L | TNFSF5/CD40L | (T cell dependent) | | | |  | |  | |  |
| activation-induced cytidine deaminase | AICDA |  | | | | [20] [21] | |  | |  |
| C epsilon germline-transcript | S-region IgE |  |  | |  | |  | |  | |
|  |  |  |  | | |  | |  | |  |
|  |  |  | | | |  | |  | |  |
|  |  |  |  | | |  | |  | |  |

1. Takeda K, Kaisho T, Akira S (2003) Toll-like receptors. Annu Rev Immunol 21: 335-376.

2. Takeda K, Akira S (2005) Toll-like receptors in innate immunity. Int Immunol 17: 1-14.

3. Kawai T, Akira S (2010) The role of pattern-recognition receptors in innate immunity: update on Toll-like receptors. Nat Immunol 11: 373-384.

4. Colonna M (2003) TREMs in the immune system and beyond. Nat Rev Immunol 3: 445-453.

5. Akira S, Uematsu S, Takeuchi O (2006) Pathogen recognition and innate immunity. Cell 124: 783-801.

6. Chamaillard M, Hashimoto M, Horie Y, Masumoto J, Qiu S, et al. (2003) An essential role for NOD1 in host recognition of bacterial peptidoglycan containing diaminopimelic acid. Nat Immunol 4: 702-707.

7. Liew FY, Xu D, Brint EK, O'Neill LA (2005) Negative regulation of toll-like receptor-mediated immune responses. Nat Rev Immunol 5: 446-458.

8. Flannery S, Bowie AG (2010) The interleukin-1 receptor-associated kinases: critical regulators of innate immune signalling. Biochem Pharmacol 80: 1981-1991.

9. Frei R, Steinle J, Birchler T, Loeliger S, Roduit C, et al. (2010) MHC class II molecules enhance Toll-like receptor mediated innate immune responses. PLoS One 5: e8808.

10. Mansell A, Smith R, Doyle SL, Gray P, Fenner JE, et al. (2006) Suppressor of cytokine signaling 1 negatively regulates Toll-like receptor signaling by mediating Mal degradation. Nat Immunol 7: 148-155.

11. Kobayashi K, Hernandez LD, Galan JE, Janeway CA, Jr., Medzhitov R, et al. (2002) IRAK-M is a negative regulator of Toll-like receptor signaling. Cell 110: 191-202.

12. Zhang G, Ghosh S (2002) Negative regulation of toll-like receptor-mediated signaling by Tollip. J Biol Chem 277: 7059-7065.

13. Chuang TH, Ulevitch RJ (2004) Triad3A, an E3 ubiquitin-protein ligase regulating Toll-like receptors. Nat Immunol 5: 495-502.

14. Wald D, Qin J, Zhao Z, Qian Y, Naramura M, et al. (2003) SIGIRR, a negative regulator of Toll-like receptor-interleukin 1 receptor signaling. Nat Immunol 4: 920-927.

15. Barton GM, Medzhitov R (2004) Toll signaling: RIPping off the TNF pathway. Nat Immunol 5: 472-474.

16. Szabo SJ, Kim ST, Costa GL, Zhang X, Fathman CG, et al. (2000) A novel transcription factor, T-bet, directs Th1 lineage commitment. Cell 100: 655-669.

17. Zheng W, Flavell RA (1997) The transcription factor GATA-3 is necessary and sufficient for Th2 cytokine gene expression in CD4 T cells. Cell 89: 587-596.

18. Fontenot JD, Gavin MA, Rudensky AY (2003) Foxp3 programs the development and function of CD4+CD25+ regulatory T cells. Nat Immunol 4: 330-336.

19. Akdis M, Burgler S, Crameri R, Eiwegger T, Fujita H, et al. (2011) Interleukins, from 1 to 37, and interferon-gamma: receptors, functions, and roles in diseases. J Allergy Clin Immunol 127: 701-721 e701-770.

20. Chaudhuri J, Alt FW (2004) Class-switch recombination: interplay of transcription, DNA deamination and DNA repair. Nat Rev Immunol 4: 541-552.

21. Ng LG, Mackay CR, Mackay F (2005) The BAFF/APRIL system: life beyond B lymphocytes. Mol Immunol 42: 763-772.

**Table S2. Association of farm life with gene expression of TLR co-receptors, molecules of the TLR signaling cascade, and of PRR**

|  | **GMR** | **95%CI** |  | **p-value** | **p-value after** |
| --- | --- | --- | --- | --- | --- |
|  |  |  |  |  | **mutliple testing** |
| NOD1 | 0.95 | 0.79 | 1.14 | 0.611 |  |
| NOD2 | 0.97 | 0.8 | 1.19 | 0.789 |  |
|  |  |  |  |  |  |
| TREM1 | 0.89 | 0.72 | 1.1 | 0.295 |  |
| LY96 | 0.87 | 0.71 | 1.06 | 0.158 |  |
|  |  |  |  |  |  |
| MYD88 | 0.85 | 0.73 | 0.99 | 0.035 | 0.101 |
| TIRAP | 1.2 | 0.97 | 1.48 | 0.086 |  |
| **TRIF** | **1.21** | **1.04** | **1.41** | **0.015** | **0.056** |
| TRAF6 | 1.05 | 0.91 | 1.21 | 0.493 |  |
| **IRAK1** | **1.17** | **1.07** | **1.29** | **0.001** | **0.052** |
| **IRAK2** | **1.89** | **1.56** | **2.29** | **0.001** | **0.026** |
| **IRAK4** | **0.83** | **0.73** | **0.96** | **0.01** | **0.047** |
| PRKR | 1.16 | 1 | 1.35 | 0.056 |  |
| **RIPK1** | **1.27** | **1.13** | **1.44** | **0.001** | **0.017** |
| RIPK2 | 0.89 | 0.77 | 1.03 | 0.126 |  |
| **TBK1** | **1.17** | **1.04** | **1.33** | **0.012** | **0.052** |
| **HLA-DRA** | **1.54** | **1.29** | **1.84** | **0.001** | **0.013** |
|  |  |  |  |  |  |
| **SOCS1** | **1.19** | **1.03** | **1.39** | **0.021** | **0.068** |
| SOCS2 | 1.85 | 0.64 | 5.35 | 0.256 |  |
| SOCS3 | 0.85 | 0.67 | 1.08 | 0.183 |  |
| **SOCS4** | **1.17** | **1.04** | **1.31** | **0.008** | **0.042** |
| SOCS5 | 1.21 | 0.81 | 1.82 | 0.354 |  |
| SOCS6 | 1.03 | 0.76 | 1.4 | 0.842 |  |
| SOCS7 | 1.1 | 0.94 | 1.29 | 0.237 |  |
| IRAK3 | 1.11 | 0.97 | 1.27 | 0.13 |  |
| TOLLIP | 1.05 | 0.97 | 1.14 | 0.246 |  |
| SARM1^1^ | 1.01 | 0.9 | 1.13 | 0.928 |  |
| TRIAD3 | 1.1 | 1 | 1.22 | 0.058 |  |
| SIGIRR | 0.95 | 0.86 | 1.06 | 0.38 |  |
| RIPK3 | 0.85 | 0.73 | 1 | 0.049 |  |

 *adjusted for sex, age, mother atopic sensitization and father atopic sensitization

GMR, geometric mean in farmers’ children compared with non-farmers’ children

^1^ Binomial values

Boldface values: p<0.1

**Table S3. Association of PRR, TLR co-receptors, and molecules of the TLR signaling cascade with asthma, rhinoconjunctivitis, CSR to IgE, and total or allergen-specific IgE**

|  | **Asthma** | |  |  | | | **Rhinoconjunctivitis** | | |  | | |
| --- | --- | --- | --- | --- | --- | --- | --- | --- | --- | --- | --- | --- |
|  | **OR** | **95%CI** |  | **P-value** | **p-value after** |  | **OR** | **95%CI** |  | **P-value** | **p-value after** |  |
|  |  |  |  |  | **mutliple testing** |  |  |  |  |  | **mutliple testing** |  |
| TLR1 | 0.87 | 0.51 | 1.5 | 0.623 |  |  | 0.94 | 0.5 | 1.78 | 0.849 |  |  |
| TLR2 | 0.67 | 0.35 | 1.28 | 0.223 |  |  | 0.97 | 0.49 | 1.92 | 0.923 |  |  |
| TLR3 | 0.96 | 0.86 | 1.08 | 0.492 |  |  | 1.08 | 0.9 | 1.3 | 0.385 |  |  |
| TLR4 | 0.87 | 0.46 | 1.66 | 0.68 |  |  | 0.58 | 0.29 | 1.18 | 0.133 |  |  |
| TLR5 | 1.12 | 0.73 | 1.72 | 0.591 |  |  | 1.29 | 0.77 | 2.17 | 0.335 |  |  |
| TLR6 | 0.99 | 0.54 | 1.82 | 0.973 |  |  | 0.65 | 0.32 | 1.32 | 0.23 |  |  |
| TLR7 | 0.74 | 0.42 | 1.32 | 0.308 |  |  | 1.07 | 0.58 | 1.96 | 0.829 |  |  |
| TLR8_1^2^ | 0.97 | 0.57 | 1.64 | 0.904 |  |  | 0.84 | 0.47 | 1.51 | 0.561 |  |  |
| TLR8_2^2^ | 0.74 | 0.48 | 1.16 | 0.189 |  |  | 0.69 | 0.42 | 1.13 | 0.14 |  |  |
| TLR9 | 0.8 | 0.47 | 1.34 | 0.396 |  |  | 0.6 | 0.33 | 1.07 | 0.085 |  |  |
| TLR10 | 0.88 | 0.69 | 1.12 | 0.313 |  |  | 0.86 | 0.67 | 1.11 | 0.251 |  |  |
| CD14 | 0.49 | 0.2 | 1.21 | 0.123 |  |  | 0.58 | 0.22 | 1.55 | 0.277 |  |  |
| TREM1 | 0.93 | 0.57 | 1.5 | 0.751 |  |  | 0.66 | 0.39 | 1.11 | 0.117 |  |  |
| NOD1 | 0.77 | 0.5 | 1.19 | 0.239 |  |  | 1.3 | 0.66 | 2.6 | 0.45 |  |  |
| NOD2 | 0.92 | 0.55 | 1.52 | 0.74 |  |  | 0.72 | 0.41 | 1.27 | 0.254 |  |  |
| LY96 | 0.66 | 0.38 | 1.14 | 0.133 |  |  | 0.64 | 0.34 | 1.19 | 0.161 |  |  |
|  |  |  |  |  |  |  |  |  |  |  |  |  |
| MYD88 | 0.7 | 0.35 | 1.4 | 0.316 |  |  | 0.81 | 0.37 | 1.75 | 0.592 |  |  |
| TIRAP | 1.5 | 0.82 | 2.76 | 0.186 |  |  | 0.78 | 0.49 | 1.24 | 0.291 |  |  |
| TRIF | 1.08 | 0.53 | 2.2 | 0.831 |  |  | 0.75 | 0.33 | 1.66 | 0.474 |  |  |
| TRAF6 | 0.95 | 0.43 | 2.08 | 0.9 |  |  | 0.59 | 0.25 | 1.39 | 0.231 |  |  |
| IRAK1 | 0.27 | 0.09 | 0.8 | 0.018 | 0.378 |  | 1.97 | 0.58 | 6.66 | 0.275 |  |  |
| IRAK2 | 0.50 | 0.26 | 0.97 | 0.039 | 0.351 |  | 0.75 | 0.37 | 1.52 | 0.432 |  |  |
| IRAK4 | 0.96 | 0.42 | 2.16 | 0.915 |  |  | 0.95 | 0.38 | 2.41 | 0.918 |  |  |
| PRKR | 0.87 | 0.41 | 1.85 | 0.718 |  |  | 1.11 | 0.49 | 2.53 | 0.805 |  |  |
| RIPK1 | 0.36 | 0.14 | 0.94 | 0.037 | 0.466 |  | 0.51 | 0.18 | 1.46 | 0.209 |  |  |
| RIPK2 | 1.16 | 0.58 | 2.32 | 0.676 |  |  | 0.99 | 0.44 | 2.19 | 0.975 |  |  |
| TBK1 | 0.46 | 0.17 | 1.2 | 0.113 |  |  | 0.67 | 0.24 | 1.91 | 0.458 |  |  |
| HLA-DRA | 0.91 | 0.47 | 1.76 | 0.779 |  |  | 0.82 | 0.39 | 1.72 | 0.595 |  |  |
|  |  |  |  |  |  |  |  |  |  |  |  |  |
| SOCS1 | 0.88 | 0.42 | 1.84 | 0.744 |  |  | 0.75 | 0.31 | 1.79 | 0.514 |  |  |
| SOCS2 | 1.44 | 1.02 | 2.03 | 0.038 | 0.399 |  | 1.78 | 1.18 | 2.67 | 0.006 | 0.378 |  |
| SOCS3 | 1.1 | 0.71 | 1.7 | 0.673 |  |  | 0.91 | 0.56 | 1.5 | 0.718 |  |  |
| SOCS4 | 0.75 | 0.31 | 1.76 | 0.504 |  |  | 0.77 | 0.29 | 2.06 | 0.606 |  |  |
| SOCS5 | 1.04 | 0.85 | 1.28 | 0.704 |  |  | 0.98 | 0.8 | 1.21 | 0.872 |  |  |
| SOCS6 | 0.95 | 0.7 | 1.29 | 0.753 |  |  | 0.76 | 0.57 | 1.01 | 0.062 |  |  |
| SOCS7 | 1.2 | 0.6 | 2.39 | 0.607 |  |  | 1.26 | 0.6 | 2.63 | 0.535 |  |  |
| IRAK3 | 0.62 | 0.25 | 1.53 | 0.303 |  |  | 0.52 | 0.19 | 1.42 | 0.202 |  |  |
| TOLLIP | 0.4 | 0.11 | 1.44 | 0.16 |  |  | 0.41 | 0.1 | 1.75 | 0.23 |  |  |
| SARM1^1^ | 1.06 | 0.43 | 2.58 | 0.91 |  |  | 1.29 | 0.47 | 3.52 | 0.616 |  |  |
| TRIAD3 | 0.82 | 0.29 | 2.36 | 0.718 |  |  | 0.87 | 0.26 | 2.84 | 0.812 |  |  |
| SIGIRR | 0.32 | 0.12 | 0.85 | 0.023 | 0.362 |  | 0.6 | 0.2 | 1.83 | 0.37 |  |  |
| RIPK3 | 0.81 | 0.47 | 1.42 | 0.464 |  |  | 1.31 | 0.59 | 2.88 | 0.509 |  |  |
|  |  |  |  |  |  |  |  |  |  |  |  |  |

|  | **Cε GLT** | | | | |  |  | **Total IgE** | | | | |
| --- | --- | --- | --- | --- | --- | --- | --- | --- | --- | --- | --- | --- |
|  | **OR** | **95%CI** |  | **P-value** | **p-value after** |  |  | **OR** | **95%CI** |  | **P-value** | **p-value after** |
|  |  |  |  |  | **mutliple testing** |  |  |  |  |  |  | **mutliple testing** |
| TLR1 | 1.02 | 0.86 | 1.21 | 0.813 |  |  | TLR1 | 1.00 | 0.81 | 1.25 | 0.982 |  |
| TLR2 | 0.83 | 0.69 | 1.00 | 0.049 | 0.193 |  | TLR2 | 1.01 | 0.9 | 1.12 | 0.922 |  |
| TLR3 | 1.03 | 0.98 | 1.08 | 0.191 |  |  | TLR3 | 0.9 | 0.71 | 1.14 | 0.384 |  |
| TLR4 | 0.80 | 0.65 | 0.97 | 0.023 | 0.111 |  | TLR4 | 1.00 | 0.95 | 1.05 | 0.977 |  |
| TLR5 | 0.93 | 0.82 | 1.06 | 0.304 |  |  | TLR5 | 0.79 | 0.64 | 0.97 | 0.024 | 0.756 |
| TLR6 | 0.91 | 0.76 | 1.09 | 0.300 |  |  | TLR6 | 0.92 | 0.78 | 1.08 | 0.312 |  |
| **TLR7** | **1.33** | **1.13** | **1.57** | **0.001** | **0.063** |  | TLR7 | 0.81 | 0.64 | 1.02 | 0.078 |  |
| TLR8_1^2^ | 0.98 | 0.83 | 1.15 | 0.785 |  |  | TLR8_1^2^ | 1.02 | 0.82 | 1.26 | 0.873 |  |
| TLR8_2^2^ | 1.07 | 0.93 | 1.23 | 0.351 |  |  | TLR8_2^2^ | 0.80 | 0.65 | 0.99 | 0.038 | 0.599 |
| **TLR9** | **1.37** | **1.16** | **1.61** | **0.001** | **0.032** |  | TLR9 | 0.93 | 0.78 | 1.12 | 0.466 |  |
| **TLR10** | **0.9** | **0.83** | **0.98** | **0.017** | **0.089** |  | TLR10 | 0.91 | 0.74 | 1.13 | 0.402 |  |
| CD14 | 0.92 | 0.7 | 1.19 | 0.52 |  |  | CD14 | 0.81 | 0.57 | 1.14 | 0.22 |  |
| TREM1 | 0.86 | 0.74 | 1 | 0.054 |  |  | TREM1 | 0.80 | 0.66 | 0.97 | 0.025 | 0.525 |
| NOD1 | 1.07 | 0.9 | 1.28 | 0.449 |  |  | NOD1 | 1.16 | 0.93 | 1.44 | 0.184 |  |
| NOD2 | 0.84 | 0.72 | 0.99 | 0.033 | 0.149 |  | NOD2 | 0.87 | 0.71 | 1.07 | 0.19 |  |
| LY96 | 0.85 | 0.73 | 1.01 | 0.058 |  |  | LY96 | 0.91 | 0.74 | 1.12 | 0.36 |  |
|  |  |  |  |  |  |  |  |  |  |  |  |  |
| MYD88 | 0.86 | 0.69 | 1.07 | 0.17 |  |  | MYD88 | 0.79 | 0.61 | 1.04 | 0.089 |  |
| TIRAP | 1.08 | 0.92 | 1.26 | 0.34 |  |  | TIRAP | 1.01 | 0.83 | 1.22 | 0.923 |  |
| TRIF | 0.99 | 0.81 | 1.22 | 0.962 |  |  | TRIF | 0.89 | 0.68 | 1.16 | 0.386 |  |
| TRAF6 | 1.09 | 0.86 | 1.37 | 0.467 |  |  | TRAF6 | 0.8 | 0.59 | 1.07 | 0.137 |  |
| IRAK1 | 1.21 | 0.86 | 1.7 | 0.278 |  |  | IRAK1 | 1.24 | 0.81 | 1.91 | 0.318 |  |
| **IRAK2** | **1.23** | **1.04** | **1.45** | **0.014** | **0.080** |  | IRAK2 | 0.91 | 0.73 | 1.13 | 0.377 |  |
| IRAK4 | 1.22 | 0.97 | 1.54 | 0.088 |  |  | IRAK4 | 1.11 | 0.83 | 1.49 | 0.477 |  |
| PRKR | 0.92 | 0.75 | 1.13 | 0.435 |  |  | PRKR | 0.94 | 0.72 | 1.23 | 0.651 |  |
| RIPK1 | 1.14 | 0.88 | 1.49 | 0.322 |  |  | RIPK1 | 0.81 | 0.58 | 1.14 | 0.229 |  |
| RIPK2 | 0.93 | 0.75 | 1.16 | 0.526 |  |  | RIPK2 | 1.15 | 0.87 | 1.51 | 0.318 |  |
| TBK1 | 1.12 | 0.87 | 1.45 | 0.380 |  |  | TBK1 | 0.88 | 0.64 | 1.21 | 0.431 |  |
| **HLA-DRA** | **1.30** | **1.08** | **1.55** | **0.005** | **0.045** |  | HLA-DRA | 0.94 | 0.73 | 1.19 | 0.586 |  |
|  |  |  |  |  |  |  |  |  |  |  |  |  |
| SOCS1 | 0.91 | 0.73 | 1.12 | 0.365 |  |  | SOCS1 | 0.98 | 0.75 | 1.29 | 0.896 |  |
| SOCS2 | 1.1 | 0.98 | 1.22 | 0.111 |  |  | SOCS2 | 1.03 | 0.99 | 1.06 | 0.199 |  |
| SOCS3 | 1.01 | 0.98 | 1.04 | 0.518 |  |  | SOCS3 | 0.89 | 0.75 | 1.05 | 0.156 |  |
| SOCS4 | 1.23 | 0.93 | 1.61 | 0.147 |  |  | SOCS4 | 1.14 | 0.81 | 1.62 | 0.45 |  |
| SOCS5 | 1.00 | 0.84 | 1.19 | 0.999 |  |  | SOCS5 | 1.03 | 0.93 | 1.15 | 0.543 |  |
| **SOCS6** | **1.14** | **1.03** | **1.27** | **0.012** | **0.095** |  | SOCS6 | 0.97 | 0.85 | 1.11 | 0.705 |  |
| SOCS7 | 1.2 | 0.98 | 1.47 | 0.077 |  |  | SOCS7 | 0.99 | 0.76 | 1.28 | 0.911 |  |
| IRAK3 | 1.1 | 0.87 | 1.39 | 0.422 |  |  | IRAK3 | 0.79 | 0.58 | 1.07 | 0.127 |  |
| TOLLIP | 1.43 | 0.98 | 2.1 | 0.064 |  |  | TOLLIP | 1.20 | 0.74 | 1.94 | 0.466 |  |
| SARM1^1^ | 1 | 0.76 | 1.32 | 0.994 |  |  | SARM1^1^ | 0.99 | 0.70 | 1.41 | 0.966 |  |
| **TRIAD3** | **1.49** | **1.09** | **2.02** | **0.012** | **0.084** |  | TRIAD3 | 0.97 | 0.65 | 1.44 | 0.875 |  |
| SIGIRR | 1.25 | 0.93 | 1.69 | 0.138 |  |  | SIGIRR | 0.91 | 0.62 | 1.33 | 0.621 |  |
| RIPK3 | 0.95 | 0.78 | 1.17 | 0.636 |  |  | RIPK3 | 1.12 | 0.87 | 1.45 | 0.385 |  |

|  | **Atopic Sensitization** | |  |  |  |
| --- | --- | --- | --- | --- | --- |
|  | **OR** | **95%CI** |  | **P-value** | **p-value after** |
|  |  |  |  |  | **mutliple testing** |
| TLR1 | 0.821 | 0.600 | 1.124 | 0.218 |  |
| TLR2 | 0.914 | 0.647 | 1.291 | 0.610 |  |
| TLR3 | 0.986 | 0.917 | 1.061 | 0.714 |  |
| TLR4 | 0.859 | 0.600 | 1.229 | 0.406 |  |
| TLR5 | 0.956 | 0.756 | 1.209 | 0.707 |  |
| TLR6 | 0.661 | 0.467 | 0.936 | 0.020 | 0.312 |
| TLR7 | 1.153 | 0.841 | 1.579 | 0.376 |  |
| TLR8_1^2^ | 0.717 | 0.526 | 0.977 | 0.035 | 0.315 |
| TLR8_2^2^ | 0.796 | 0.615 | 1.031 | 0.084 |  |
| TLR9 | 0.843 | 0.616 | 1.154 | 0.288 |  |
| TLR10 | 0.966 | 0.820 | 1.138 | 0.678 |  |
| CD14 | 0.52 | 0.32 | 0.86 | 0.011 | 0.240 |
| TREM1 | 0.78 | 0.60 | 1.02 | 0.069 |  |
| NOD1 | 1.33 | 0.95 | 1.88 | 0.100 |  |
| NOD2 | 0.75 | 0.56 | 1.02 | 0.067 |  |
| LY96 | 0.77 | 0.57 | 1.04 | 0.085 |  |
|  |  |  |  |  |  |
| MYD88 | 0.81 | 0.55 | 1.21 | 0.303 |  |
| TIRAP | 1.08 | 0.81 | 1.45 | 0.592 |  |
| TRIF | 0.88 | 0.60 | 1.29 | 0.502 |  |
| TRAF6 | 0.80 | 0.52 | 1.22 | 0.304 |  |
| IRAK1 | 0.80 | 0.44 | 1.49 | 0.488 |  |
| IRAK2 | 0.78 | 0.57 | 1.07 | 0.123 |  |
| IRAK4 | 1.14 | 0.72 | 1.80 | 0.575 |  |
| PRKR | 1.14 | 0.72 | 1.80 | 0.575 |  |
| RIPK1 | 0.46 | 0.27 | 0.78 | 0.004 | 0.280 |
| RIPK2 | 1.03 | 0.70 | 1.52 | 0.882 |  |
| TBK1 | 0.59 | 0.36 | 0.97 | 0.039 | 0.307 |
| HLA-DRA | 1.11 | 0.79 | 1.56 | 0.547 |  |
|  |  |  |  |  |  |
| SOCS1 | 0.93 | 0.63 | 1.38 | 0.719 |  |
| SOCS2 | 1.01 | 0.95 | 1.07 | 0.759 |  |
| SOCS3 | 0.87 | 0.69 | 1.09 | 0.219 |  |
| SOCS4 | 0.87 | 0.53 | 1.43 | 0.579 |  |
| SOCS5 | 0.97 | 0.84 | 1.11 | 0.671 |  |
| SOCS6 | 0.98 | 0.81 | 1.19 | 0.863 |  |
| SOCS7 | 1.04 | 0.72 | 1.52 | 0.823 |  |
| IRAK3 | 0.60 | 0.37 | 0.96 | 0.033 | 0.345 |
| TOLLIP | 1.09 | 0.54 | 2.19 | 0.812 |  |
| SARM1^1^ | 0.91 | 0.55 | 1.52 | 0.727 |  |
| TRIAD3 | 0.86 | 0.48 | 1.54 | 0.606 |  |
| SIGIRR | 0.97 | 0.56 | 1.67 | 0.903 |  |
| RIPK3 | 1.04 | 0.71 | 1.53 | 0.838 |  |

*adjusted for farmer, sex, age, mother atopic sensitization and father atopic sensitization

OR, odds ratio of farmers’ children compared with non-farmers’ children

^1^ Binomial values

^2^ Isoform 1 (TLR8.1) encodes the longer isoform, which has an extended N-terminus compared with isoform 2 (TLR8.2)

Boldface values: p<0.1

**Table S4. Association of gene expression of markers of T helper cell subtypes with asthma, rhinoconjunctivitis, CSR to IgE, and total or allergen-specific** IgE

|  | **Asthma** |  |  |  |  |
| --- | --- | --- | --- | --- | --- |
|  | OR* | 95%CI |  | p-value | p-value multiple testing |
| T-bet | 0.82 | 0.44 | 1.56 | 0.55 |  |
| CCR5 | 1.06 | 0.83 | 1.34 | 0.64 |  |
|  |  |  |  |  |  |
| GATA3 | 0.86 | 0.44 | 1.67 | 0.65 |  |
| CCR3 | 1.74 | 1.19 | 2.54 | 0.004 | 0.252 |
| CCR4 | 0.98 | 0.62 | 1.54 | 0.92 |  |
|  |  |  |  |  |  |
| FOXP3 | 0.95 | 0.49 | 1.87 | 0.89 |  |

|  | **Rhinoconjunctivitis** |  |  |  |  |
| --- | --- | --- | --- | --- | --- |
|  | OR* | 95%CI |  | p-value | p-value multiple testing |
| T-bet | 1.36 | 0.66 | 2.82 | 0.4 |  |
| CCR5 | 0.91 | 0.76 | 1.09 | 0.299 |  |
|  |  |  |  |  |  |
| GATA3 | 0.53 | 0.24 | 1.17 | 0.115 |  |
| CCR3 | 1.63 | 1.08 | 2.46 | 0.021 | 0.662 |
| CCR4 | 0.76 | 0.43 | 1.35 | 0.346 |  |
|  |  |  |  |  |  |
| FOXP3 | 0.95 | 0.45 | 2 | 0.896 |  |

|  | **Cε GLT** |  |  |  |  |
| --- | --- | --- | --- | --- | --- |
|  | GMR* | 95%CI |  | p-value | p-value multiple testing |
| **T-bet** | **1.33** | **1.10** | **1.61** | **0.004** | **0.042** |
| CCR5 | 0.97 | 0.83 | 1.14 | 0.735 |  |
|  |  |  |  |  |  |
| **GATA3** | **1.56** | **1.29** | **1.88** | **0.001** | **0.021** |
| CCR3 | 0.94 | 0.83 | 1.06 | 0.29 |  |
| CCR4 | 1.03 | 0.9 | 1.19 | 0.646 |  |
|  |  |  |  |  |  |
| FOXP3 | 1.25 | 1.02 | 1.54 | 0.034 | 0.143 |

|  | **total IgE** |  |  |  |  |
| --- | --- | --- | --- | --- | --- |
|  | GMR* | 95%CI |  | p-value | p-value multiple testing |
| T-bet | 1.14 | 0.89 | 1.46 | 0.315 |  |
| CCR5 | 0.99 | 0.91 | 1.07 | 0.715 |  |
|  |  |  |  |  |  |
| GATA3 | 1.03 | 0.81 | 1.31 | 0.81 |  |
| CCR3 | 1.23 | 1.06 | 1.42 | 0.006 | 0.378 |
| CCR4 | 1.07 | 0.9 | 1.28 | 0.429 |  |
|  |  |  |  |  |  |
| FOXP3 | 1.01 | 0.77 | 1.31 | 0.946 |  |

|  | **Atopic Sensitization** | | |  |  |
| --- | --- | --- | --- | --- | --- |
|  | GMR* | 95%CI |  | p-value | p-value multiple testing |
| T-bet | 1.44 | 1.01 | 2.07 | 0.047 | 0.293 |
| CCR5 | 1.03 | 0.91 | 1.17 | 0.631 |  |
|  |  |  |  |  |  |
| GATA3 | 1.16 | 0.81 | 1.65 | 0.419 |  |
| CCR3 | 1.25 | 1.01 | 1.56 | 0.043 | 0.304 |
| CCR4 | 1.05 | 0.82 | 1.35 | 0.680 |  |
|  |  |  |  |  |  |
| FOXP3 | 1.54 | 1.04 | 2.27 | 0.032 | 0.399 |

*adjusted for farmer, sex, age, mother atopic sensitization and father atopic sensitization

OR, odds ratio of farmers’ children compared with non-farmers’ children

GMR, geometric mean in farmers’ children compared with non farmers’ children

Boldface values: p<0.1

**Table S5. Association of gene expression of cytokines with asthma, rhinoconjunctivitis, CSR to IgE, and total or allergen-specific IgE**

|  | **Asthma** |  |  |  |  |
| --- | --- | --- | --- | --- | --- |
|  | **OR*** | **95%CI** |  | **p-value** | **p-value multiple testing** |
| IL12α | 0.96 | 0.74 | 1.24 | 0.76 |  |
| IFN-γ | 0.98 | 0.78 | 1.22 | 0.83 |  |
| IL4^1^ | 0.45 | 0.17 | 1.15 | 0.10 |  |
| IL10 | 0.98 | 0.85 | 1.12 | 0.74 |  |
| TGF-β^1^ | 0.5 | 0.17 | 1.52 | 0.22 |  |
| IL1β | 0.67 | 0.45 | 1 | 0.05 |  |
| IL6 | 0.99 | 0.86 | 1.13 | 0.83 |  |
| IL8 | 1.16 | 0.74 | 1.82 | 0.51 |  |
| IL18 | 0.55 | 0.36 | 0.86 | 0.01 | 0.315 |
| IL21^1^ | 1.29 | 0.5 | 3.34 | 0.60 |  |
| TNF-α | 0.83 | 0.42 | 1.62 | 0.58 |  |

|  | **Rhinoconjunctivitis** |  |  |  |  |
| --- | --- | --- | --- | --- | --- |
|  | **OR*** | **95%CI** |  | **p-value** | **p-value multiple testing** |
| IL12α | 1.18 | 0.78 | 1.77 | 0.436 |  |
| IFN-γ | 0.92 | 0.73 | 1.16 | 0.476 |  |
| IL4^1^ | 0.85 | 0.31 | 2.34 | 0.759 |  |
| IL10 | 0.99 | 0.84 | 1.17 | 0.917 |  |
| TGF-β^1^ | 0.67 | 0.2 | 2.21 | 0.507 |  |
| IL1β | 0.61 | 0.39 | 0.97 | 0.035 |  |
| IL6 | 0.98 | 0.84 | 1.15 | 0.795 |  |
| IL8 | 0.9 | 0.59 | 1.37 | 0.637 |  |
| IL18 | 0.77 | 0.47 | 1.27 | 0.313 |  |
| IL21^1^ | 0.25 | 0.05 | 1.18 | 0.079 |  |
| TNF-α | 0.55 | 0.25 | 1.19 | 0.126 |  |

|  | **Cε GLT** |  |  |  |  |
| --- | --- | --- | --- | --- | --- |
|  | **GMR*** | **95%CI** |  | **p-value** | **p-value multiple testing** |
| IL12α | 1.13 | 0.98 | 1.31 | 0.087 |  |
| IFN-γ | 0.99 | 0.93 | 1.06 | 0.836 |  |
| IL4^1^ | 1.00 | 0.76 | 1.33 | 0.986 |  |
| IL10 | 0.99 | 0.94 | 1.04 | 0.655 |  |
| TGF-β^1^ | 1.28 | 0.94 | 1.74 | 0.113 |  |
| IL1β | 1.02 | 0.92 | 1.13 | 0.714 |  |
| IL6 | 1.03 | 0.98 | 1.08 | 0.204 |  |
| IL8 | 1.03 | 0.93 | 1.15 | 0.564 |  |
| IL18 | 1.03 | 0.89 | 1.19 | 0.712 |  |
| IL21^1^ | 0.84 | 0.61 | 1.16 | 0.293 |  |
| **TNF-α** | **1.38** | **1.14** | **1.67** | **0.001** | **0.016** |

|  | **total IgE** |  |  |  |  |
| --- | --- | --- | --- | --- | --- |
|  | **GMR*** | **95%CI** |  | **p-value** | **p-value multiple testing** |
| IL12α | 0.99 | 0.89 | 1.1 | 0.822 |  |
| IFN-γ | 1.00 | 0.92 | 1.09 | 0.999 |  |
| IL4^1^ | 1.09 | 0.76 | 1.55 | 0.655 |  |
| IL10 | 0.97 | 0.91 | 1.03 | 0.284 |  |
| TGF-β^1^ | 1.00 | 0.68 | 1.49 | 0.981 |  |
| IL1β | 0.98 | 0.85 | 1.12 | 0.723 |  |
| IL6 | 1.00 | 0.95 | 1.06 | 0.925 |  |
| IL8 | 0.9 | 0.79 | 1.02 | 0.109 |  |
| IL18 | 0.99 | 0.82 | 1.19 | 0.913 |  |
| IL21^1^ | 1.04 | 0.69 | 1.56 | 0.848 |  |
| TNF-α | 0.92 | 0.72 | 1.18 | 0.523 |  |

|  | **Atopic Sensitization** | |  |  |  |
| --- | --- | --- | --- | --- | --- |
|  | **OR*** | **95%CI** |  | **p-value** | **p-value multiple testing** |
| IL12α | 0.93 | 0.80 | 1.07 | 0.314 |  |
| IFN-γ | 1.01 | 0.89 | 1.14 | 0.887 |  |
| IL4^1^ | 0.72 | 0.43 | 1.21 | 0.219 |  |
| IL10 | 0.98 | 0.90 | 1.07 | 0.644 |  |
| TGF-β^1^ | 0.84 | 0.47 | 1.48 | 0.537 |  |
| IL1β | 0.98 | 0.80 | 1.19 | 0.812 |  |
| IL6 | 1.08 | 0.99 | 1.18 | 0.100 |  |
| IL8 | 0.74 | 0.60 | 0.92 | 0.006 | 0.177 |
| IL18 | 0.96 | 0.73 | 1.26 | 0.783 |  |
| IL21^1^ | 0.98 | 0.54 | 1.77 | 0.949 |  |
| TNF-α | 1.00 | 0.70 | 1.43 | 0.994 |  |
|  | |  |  |  |  |

*adjusted for farmer sex, age, mother atopic sensitization and father atopic sensitization

OR, odds ratio of farmers’ children compared with non-farmers’ children

GMR, geometric mean in farmers’ children compared with non farmers’ children

^1^ Binomial values

**Table S6.** Association of farm life with gene expression of APRIL, BAFF, CD40L, and AICDA

|  | GMR* | 95%CI |  | p-value |
| --- | --- | --- | --- | --- |
| APRIL | 0.91 | 0.79 | 1.05 | 0.19 |
| BAFF | 1.09 | 0.91 | 1.31 | 0.328 |
| AICDA^1^ | 1.05 | 0.94 | 1.17 | 0.379 |
| CD40L | 1.1 | 0.93 | 1.29 | 0.253 |

*adjusted for farmer sex, age, mother atopic sensitization and father atopic sensitization

GMR, geometric mean in farmers’ children compared with non farmers’ children

^1^ Binomial values

***Table S7. Association of gene expression of APRIL, BAFF, CD40L, and AICDA with asthma, rhinoconjunctivitis, CSR to IgE, and total or allergen-specific IgE***

|  | **Asthma** |  |  |  |  |
| --- | --- | --- | --- | --- | --- |
|  | **OR*** | **95%CI** |  | **p-value** | **p-value multiple testing** |
| APRIL | 0.96 | 0.44 | 2.06 | 0.91 |  |
| BAFF | 0.73 | 0.38 | 1.39 | 0.34 |  |
| AICDA^1^ | 0.76 | 0.28 | 2.1 | 0.6 |  |
| CD40L | 0.92 | 0.47 | 1.81 | 0.81 |  |

|  | **Rhinoconjunctivitis** |  |  |  |  |
| --- | --- | --- | --- | --- | --- |
|  | **OR*** | **95%CI** |  | **p-value** | **p-value multiple testing** |
| APRIL | 0.92 | 0.38 | 2.25 | 0.861 |  |
| BAFF | 0.86 | 0.42 | 1.78 | 0.693 |  |
| AICDA^1^ | 1.06 | 0.36 | 3.07 | 0.92 |  |
| CD40L | 0.51 | 0.23 | 1.15 | 0.105 |  |

|  | **Cε GLT** |  |  |  |  |
| --- | --- | --- | --- | --- | --- |
|  | **GMR*** | **95%CI** |  | **p-value** | **p-value multiple testing** |
| APRIL | 1.14 | 0.91 | 1.43 | 0.248 |  |
| BAFF | 0.96 | 0.8 | 1.14 | 0.624 |  |
| **AICDA^1^** | **0.69** | **0.51** | **0.92** | **0.012** | **0.076** |
| **CD40L** | **1.46** | **1.2** | **1.77** | **0.001** | **0.013** |

|  | **total IgE** |  |  |  |  |
| --- | --- | --- | --- | --- | --- |
|  | **GMR*** | **95%CI** |  | **p-value** | **p-value multiple testing** |
| APRIL | 0.89 | 0.67 | 1.18 | 0.427 |  |
| BAFF | 0.95 | 0.75 | 1.19 | 0.632 |  |
| AICDA^1^ | 1.04 | 0.71 | 1.53 | 0.826 |  |
| CD40L | 1.01 | 0.78 | 1.31 | 0.93 |  |

|  | **Atopic Sensitization** | | |  |  |
| --- | --- | --- | --- | --- | --- |
|  | **OR*** | **95%CI** |  | **p-value** | **p-value multiple testing** |
| APRIL | 1.066 | 0.698 | 1.628 | 0.769 |  |
| BAFF | 0.769 | 0.544 | 1.087 | 0.136 |  |
| AICDA^1^ | 0.999 | 0.575 | 1.738 | 0.998 |  |
| CD40L | 1.226 | 0.851 | 1.766 | 0.273 |  |

*adjusted for farmer sex, age, mother atopic sensitization and father atopic sensitization

^1^ Binomial values

OR, odds ratio of farmers’ children compared with non-farmers’ children

GMR, geometric mean in farmers’ children compared with non farmers’ children
